# Structural Features and Mechanical Properties of Hydrogels Based on PVP Copolymers, Obtained in the Presence of a Solvent

**DOI:** 10.3390/gels11121008

**Published:** 2025-12-13

**Authors:** Oleksandr Grytsenko, Petro Pukach, Myroslava Vovk, Nataliia Baran

**Affiliations:** 1 Institute of Chemistry and Chemical Technologies, Lviv Polytechnic National University, 12 St. Bandera Str., 79013 Lviv, Ukraine; oleksandr.m.grytsenko@lpnu.ua (O.G.); nataliia.m.baran@lpnu.ua (N.B.); 2 Institute of Applied Mathematics and Fundamental Sciences, Lviv Polytechnic National University, 12 Bandera Str., 79013 Lviv, Ukraine; myroslava.i.vovk@lpnu.ua

**Keywords:** polyvinylpyrrolidone, 2-hydroxyethylmethacrylate, solvent, graft copolymer, crosslinked polymer, polymer network, copolymerization, hydrogel, mechanical properties

## Abstract

The paper analyses the effect of the solvent amount and nature on the structure and mechanical properties of hydrogels based on copolymers of 2-hydroxyethylmethacrylate (HEMA) with polyvinylpyrrolidone (PVP). The synthesis of pHEMA-gr-PVP copolymers was carried out by the copolymerization method in the presence of metal ions of variable oxidation states in solvents with various nature: water, dimethyl sulfoxide (DMSO), diethylene glycol (DEG), and cyclohexanol (HOCy). The structure of the copolymers was evaluated by the PVP grafting efficiency, its actual content in the copolymer, and the molecular weight between crosslinks (M_C_). Taking the example of water, an increase in the solvent content up to 50 mass parts causes an increase in the efficiency of PVP grafting, which occurs due to enhanced macromolecule mobility through the dilution of the starting composition, hence the decrease in its viscosity. It was established that the nature of the solvent significantly affects the crosslinking density of the polymer network in the series H_2_O, DEG, DMSO, HOCy, an increase in the M_C_ is observed causing a decrease in the hardness and elasticity of hydrogels and an increase in their water-retention capacity and swelling coefficient. The obtained results prove the possibility of targeted regulation within wide limits of the structure and properties of hydrogels based on pHEMA-gr-PVP copolymers through control of polymerization conditions (selection of the type and concentration of solvent).

## 1. Introduction

Polymer hydrogels are among the most promising materials in the modern biomedical field [1,2,3]. The unique property of hydrogel materials to sorb and retain large amounts of solvent while maintaining their structure makes them ideal materials for tissue engineering [4,5], ophthalmology [6,7], manufacturing drug delivery systems [8,9], multifunctional wound dressings [10,11,12], sensors [13,14], etc.

However, despite their high biocompatibility, permeability to oxygen and nutrients, and elasticity, most hydrogels obtained by traditional methods are characterized by insufficient mechanical strength or sorption capacity. Hydrogel materials, depending on their purpose and operating conditions must meet different requirements for their properties. In particular, low mechanical strength and sorption–diffusion ability limit the use of hydrogels in conditions accompanied by increased pressure and additional loads, for example, in contact lenses, wound dressings, dialysis membranes, and scaffolds for tissue engineering [15]. Important requirements for the performance characteristics of these products are a combination of high physical and mechanical properties as well as sorption–diffusion ones. Therefore, special attention in modern science is focused on the hydrogels that exhibit improved properties, which is critically important for their application [16,17].

An increase in the strength of hydrogels can be achieved, for instance, by methods involving the formation of double networks [15,18,19], ionic bonds [20], or by incorporating nanoparticles [21,22]. The sorption–diffusion capacity can be increased by introducing additional hydrophilic functional groups (copolymers or polymers) into the polymer structure or by increasing the porosity of the hydrogel.

One of the most effective ways to modify and obtain hydrogels with predicted properties is copolymerization. In this context, hydrogels based on copolymers of 2-hydroxyethylmethacrylate (HEMA) and polyvinylpyrrolidone (PVP) draw a particular interest. Nowadays, the prospects for the use of hydrogels based on copolymers of HEMA with PVP (pHEMA-gr-PVP) cover various fields of science and practice: medical dressings [23], contact lenses [24], materials for the regeneration of damaged tissues [25], as enzyme immobilization systems [26], drug delivery systems [27], sensors [28], electrical elements of optical systems [29], etc.

2-hydroxyethylmethacrylate is a classic monomer used to obtain transparent, chemically and thermally stable, biologically inert polyHEMA-hydrogels, with satisfactory physical-mechanical properties, which are traditionally used for the manufacture of contact lenses and implants [30,31]. The hydroxyl and carbonyl groups of HEMA enable to form hydrogen bonds with water molecules. Hydrophobic methyl groups ensure the hydrolytic stability and strength properties of the hydrogel materials [32].

On the other hand, hydrogels made from pure polyHEMA have relatively low sorption–diffusion characteristics for oxygen, water, and other solvents. In order to solve this problem, hydrophilic elements are introduced into the polymer matrix of polyHEMA [33]. As an example, copolymerization of HEMA with vinylpyrrolidone [32] or PVP [34] can be carried out. The water-soluble polymer PVP is known for its high hydrophilicity and non-toxicity, acting as an effective property modifier [35]. pHEMA-gr-PVP copolymers demonstrate a combination of mechanical stability, increased hydrophilicity, improved adhesion, and compatibility with biological tissues [34]. Polymerization in the presence of metal ions of variable oxidation states offers new possibilities in the synthesis of pHEMA-gr-PVP copolymers [36,37].

Studies confirm that the operational and technological properties of pHEMA-gr-PVP copolymers depend mainly on the ratio of HEMA to PVP in the initial composition and the structural parameters of the polymer network, with the fundamental one being the molecular weight between crosslinks in the polymer network [38,39]. However, only adjusting the composition formulation is insufficient to predict the properties of the final product, which requires further study of the influence of the synthesis conditions of HEMA with PVP copolymers.

Currently, studies of the processes of copolymerization of HEMA with PVP in the presence of metal ions of variable oxidation states are focused mainly on bulk polymerization [36]; however, the effect of the solvent as a polymerization medium is an important factor that has a decisive impact on the hydrogel properties.

The solvent ensures homogeneous distribution of monomers and other components in the reaction mixture, which allows to obtain a more homogeneous polymer network. Structural inhomogeneity caused by local concentration gradients can lead to anisotropy of mechanical properties [32]. The formation of inhomogeneous polymer networks with uneven crosslink distribution represents yet another significant problem [40]. Inhomogeneity in crosslink density causes anisotropy in physico-mechanical properties, potentially worsening the overall characteristics of the material [41]. The anisotropy of properties is especially evident during significant deformations, where defects and inhomogeneities of the polymer network profoundly affect the mechanical behavior of the hydrogel [42].

Experimental studies indicate a direct dependence of the mechanical properties of hydrogels on the presence of a solvent in the starting composition during polymerization [43]. In particular, the polarity of the solvent affects the formation of morphological structures of polymers and, accordingly, their mechanical properties [44].

Synthesis of hydrogels in the presence of a solvent allows the polymerization process to be controlled more precisely and therefore enables the formation of a homogeneous polymer network with a predicted crosslinking density. The solvent content can be used to regulate the concentration of monomers and initiator. This regulation affects the reaction rate and crosslinking efficiency; consequently, it directly determines the packing density of the polymer network, which changes the mechanical properties of the hydrogel [44]. The presence of a solvent opens the possibility of formation of hydrogel structures with controlled porosity. During the polymerization process, solvent molecules form pores that remain in the structure after the hydrogel drying [45]. Additional micropores improve the sorption capacity of hydrogel. At the same time, hydrogels are characterized by a regularity: an increase in porosity and sorption capacity is accompanied by a decrease in strength and vice versa.

Thus, despite significant progress in the development of functional hydrogels, in particular, based on HEMA with PVP copolymers, there is still a relevant need for systematic study of the influence of each individual ingredient of the polymer composition, especially the solvent, on the mechanical properties. Concerning a solvent as a polymerization medium, its type and concentration can radically change the structure of the hydrogel.

The aim of this study is to establish the patterns of change in the structure and properties of hydrogels based on HEMA copolymers with PVP, synthesized in the presence of a solvent, depending on its concentration and nature.

## 2. Results and Discussion

### 2.1. Research of the Structure and Formulation of HEMA with PVP Copolymers Obtained by Polymerization in the Presence of a Solvent

Studies of the regularities of HEMA polymerization in the presence of PVP described in previous works [34,38] revealed that the process is accompanied by a matrix effect with the formation of a complex with charge transfer between the components of the monomer–polymer composition (MPC). It has been established that by influencing this MPC with a small amount of active additives, in particular salts of transition metals, it is possible to change the reactivity of the compositions and influence the kinetics of the polymerization, the copolymers’ structure parameters and their properties [36], and, consequently, to expand the areas of their practical application.

The obtained regularities of (co)polymerization of PVP–(meth)acrylate compositions in the presence of metal ions of variable oxidation states reflect the influence of the compositional formulation, the nature of the catalyst, and the reaction conditions on the process rate and polymer yield. They formed the basis for further development of the technology for producing hydrogel materials based on pHEMA-gr-PVP copolymers, since the operational properties of the latter can vary widely depending on the polymerization conditions, in particular, in the presence of a solvent. Based on the analysis of the kinetic parameters of polymerization, the high reactivity of the HEMA–Me^n+^–PVP system and the activating physical interaction between the components in the starting composition through complex formation were established [34,36,38]. It has been proven that the complex formation process is strongly influenced by the presence of the solvent and its nature; water and other proton-donating solvents are favorable for the formation of PVP–Me^n+^ and HEMA–Me^n+^ complexes.

**Scheme 1:**
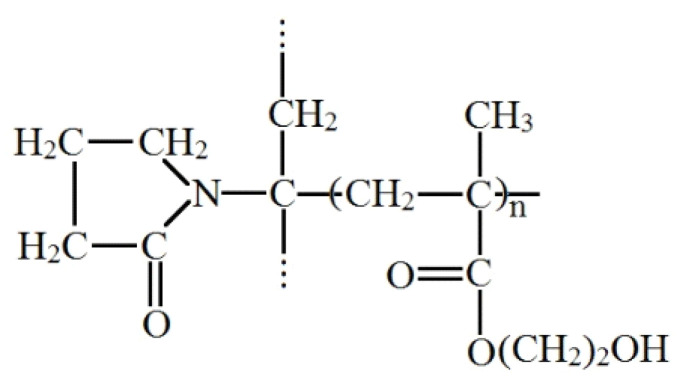
The structure of pHEMA-gr-PVP copolymer [38].

Analysis of HEMA and PVP bulk copolymerization, initiated by Me^n+^, suggests a reaction mechanism involving kinetic chain transfer to the PVP macromolecule, ultimately leading to the generation of a graft copolymer [36,38], characterized by a three-dimensional, spatially crosslinked network. In this structure, the PVP acts as the main chain, with the HEMA components forming the grafted blocks (Scheme 1).

**Figure 1:**
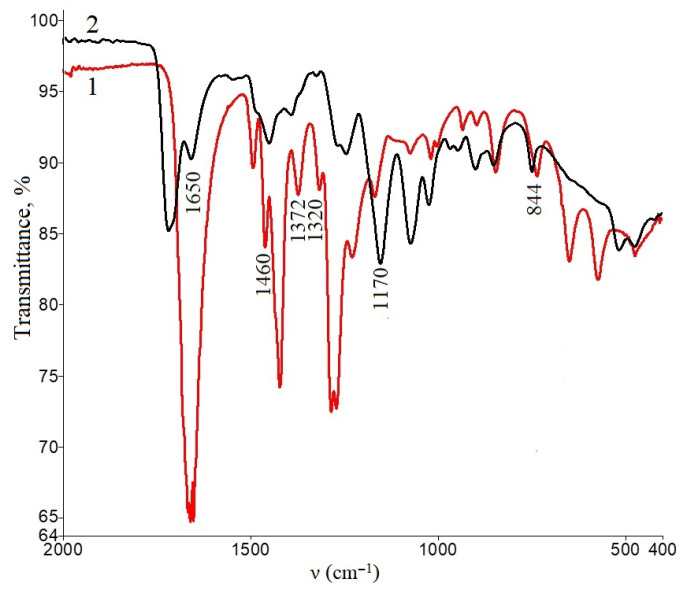
ATR FTIR spectra of PVP (1) and pHEMA-gr-PVP copolymer, obtained by polymerization in the presence of a solvent (2).

To confirm the formation of a graft copolymer obtained during polymerization in the presence of a solvent (water), IR spectra of PVP and the copolymer of HEMA with PVP (extracted by water until the unreacted PVP was completely removed) were obtained and analyzed (Figure 1).

The obtained spectra shows that characteristic PVP bands [46] in the domains of 650 cm^−1^, 844 cm^−1^, 1170 cm^−1^, 1320 cm^−1^, 1460 cm^−1^, 1650 cm^−1^ are present in the spectrum of the copolymer indicating the presence of PVP units in it.

Analyzing the obtained IR spectra, it can be noted that the intensity of the band in the domain of 1320 cm^−1^ for the copolymer (curve 2) is significantly lower compared to pure PVP (curve 1). This band characterizes the deformation oscillations of the CH group of the PVP carbon chain. Therefore, it can be assumed that the grafting of pHEMA to PVP occurs with the participation of exactly this group (Scheme 1).

Also, in the copolymer spectrum, a decrease in the band characteristic of PVP in the domain of 1372 cm^−1^ was observed. The band corresponds to the fan-shaped oscillations of the CH_2_-CH bond of polyvinylpyrrolidone and confirms the participation of tertiary carbon of PVP in the grafting process.

**Figure 2:**
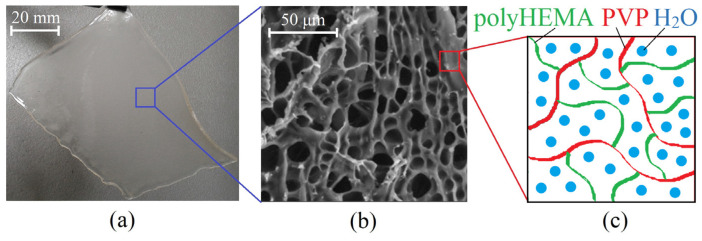
Swollen sample (**a**), SEM photograph of a porous hydrogel (**b**), and schema of the structure of copolymer (**c**) (HEMA:PVP:H_2_O = 80:20:100 mass parts).

Thus, it can be predicted that crosslinked graft copolymers of pHEMA-gr-PVP are formed as a result of the polymerization of HEMA/PVP compositions in the presence of a solvent. Images obtained using a scanning electron microscope reveal the porous structure of the hydrogel (Figure 2). The size and nature of the pores depend on the amount of washed-out, i.e., ungrafted, PVP. The porosity of the hydrogel can also be adjusted using different solvents.

The properties of a copolymer depend mainly on its structural characteristics. The structure of pHEMA-gr-PVP copolymers was characterized based on the study results of the formulation of the copolymers, the efficiency of PVP grafting, as well as the structural parameters of the polymer network: the molecular weight between crosslinks (M_C_, kg/mol) and crosslinking density (μ, mol/m^3^) which depend on the formulation of the starting composition and the nature and content of the solvent.

For hydrogels based on copolymers obtained by polymerization in bulk [38], an MPC with a maximum PVP content of up to 30 mass parts was used, which was due to technological problems—increasing the PVP content beyond this threshold significantly prolongs its dissolution time in the acrylate monomers. Furthermore, the resulting mixture exhibits elevated viscosity, making accurate dosing problematic, and contains air bubbles that are difficult to remove. In this regard, compositions with a PVP content of more than 30 mass parts are non-technological. Dilution of the composition with a solvent made it possible to increase the maximum PVP content to 50 mass parts.

**Figure 3:**
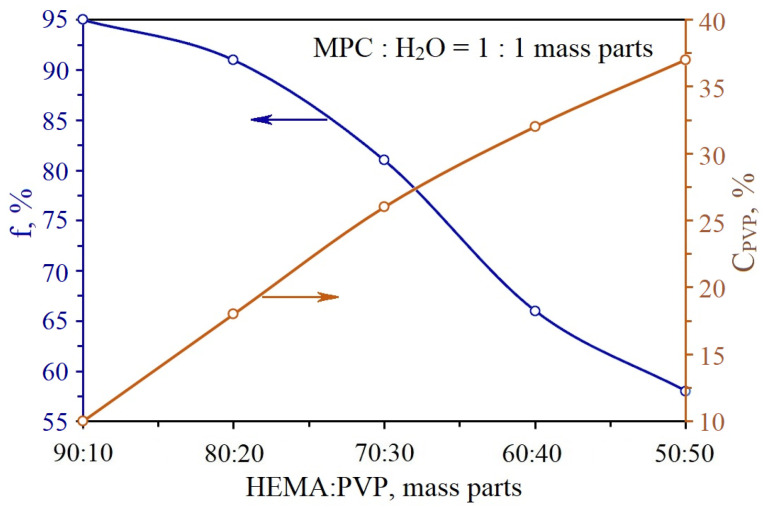
Effect of the ratio of HEMA:PVP in MPC on the formulation of copolymers.

The samples for the studies were hydrated to an equilibrium state, since products based on polymer hydrogels are used in a swollen state. The results of the composition’s effect on the structural parameters of (co)polymers are presented in Figure 3.

It has been established [36,38] that not all PVP is integrated into the final polymer network during the grafting reaction. This creates a discrepancy between the original concentration of PVP in the initial composition and its actual content in the synthesized copolymer. Therefore, it is relevant to determine the actual PVP content (C_PVP_, %) in the copolymer as a function of the HEMA:PVP formulation of the starting monomer–polymer composition. To achieve this goal, calculations were made of the PVP grafting efficiency and its final content in the final copolymer Figure 3. It was found that, as in the case of copolymers obtained in bulk [38], with an increase in the PVP content in the MPC during copolymerization in the presence of a solvent, the amount of PVP in the copolymer increases, and the grafting efficiency decreases. Table 1 shows that the highest network density (min M_C_ and max μ) is characterized by polyHEMA.

The formation of a network polymer during the polymerization of HEMA is apparently due to the presence of minor ethylene glycol dimethacrylate impurities, which play the role of crosslinking agents. With increasing PVP content in the starting composition, the M_C_ increases. The decrease in the density of the structural network of the copolymer is associated with the influence of PVP; some part of the PVP, which does not participate in the grafting reaction, is washed out during hydration, forming microvoids.

**Figure 4:**
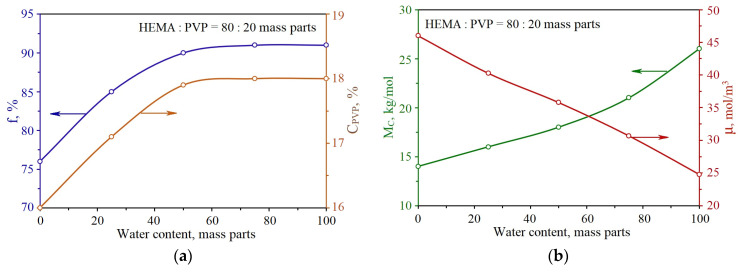
Effect of solvent amount on copolymer formulation (**a**) and structural parameters of the polymer network (**b**).

To investigate the influence of the solvent amount on the structure of copolymers, compositions with different water contents were studied (Figure 4). With an increase of the solvent amount to 50 mass parts (Figure 4a), the efficiency of PVP grafting and its content in the copolymer increase, which is obviously connected with a decrease in the viscosity of the system and an improvement in the mobility of PVP macromolecules.

The viscosity of the initial monomer–polymer composition (HEMA/PVP) without solvent or with a low solvent content is high. Under such conditions, the mobility of PVP macromolecules is significantly limited, and the active (radical) centers on the polyHEMA chains are sterically shielded. Increasing the solvent content reduces the overall viscosity of the starting composition, which accelerates the diffusion of PVP macromolecules to the reaction centers on the polyHEMA chains. This increases the probability of their effective interaction (grafting reaction). At the same time, water, which is a good solvent for both the HEMA monomer and the PVP polymer, promotes the unfolding of PVP macromolecular coils, which affects the increase in the number of PVP functional groups available for the grafting reaction.

Further increase in the solvent content to 100 mass parts does not lead to a noticeable change in grafting efficiency (f) and the copolymer formulation. At the same time, the molecular weight between crosslinks naturally increases linearly with increasing water content in the starting composition (Figure 4b).

As can be seen from the obtained results, the amount of solvent along with the composition formulation is an important factor that determines the structure of the obtained copolymers.

**Figure 5:**
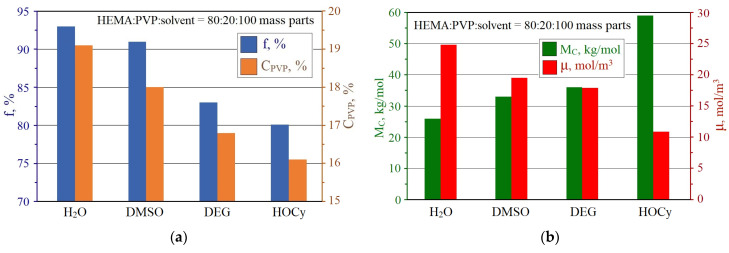
Effect of the nature of the solvent on the formulation of copolymers (**a**) and structural parameters of the polymer network (**b**).

The influence of the nature of the solvent on the structural parameters of copolymers was studied using the following examples of solvents: water, dimethyl sulfoxide (DMSO), diethylene glycol (DEG), and cyclohexanol (HOCy). For the used solvents, the grafting efficiency and the PVP content in the copolymer structure decrease in the series DEG, H_2_O, DMSO, HOCy (Figure 5a).

The obtained regularities can be explained by the differences in polarity, viscosity, and solvation capacity of the solvents used (Table 2).

DEG has a higher viscosity and provides intensive hydrogen bonding with PVP. This reduces the mobility of macromolecules and creates conditions under which radicals more easily attach to PVP. As a result, the grafting efficiency is the highest. Water, as a universal solvent, has an average solvation capacity and relatively low viscosity. This promotes chain mobility, but at the same time accelerates the homopolymerization of HEMA, which reduces the proportion of grafted PVP. DMSO is a highly polar aprotic solvent that solvates both HEMA and PVP well. In such a medium, HEMA homopolymerization prevails, and the accessibility of PVP to radicals is lower. The grafting efficiency and PVP content in the copolymer structure when using HOCy are the lowest among all solvents. HOCy is the least polar of all solvents, so it is the least effective in solvating and unfolding highly polar PVP macromolecules. The viscosity of HOCy is the highest among all solvents. High viscosity of the medium significantly limits the mobility of both HEMA monomer and PVP macromolecules. Reduced mobility of PVP decreases the probability of effective formation of PVP macroradicals necessary for the grafting reaction.

The molecular weight between crosslinks is characterized by an increase in the series H_2_O, DMSO, DEG, HOCy (Figure 5b). In water, polymerization occurs under conditions of high mobility of macromolecules, and crosslinking nodes are formed relatively frequently, and, as a result, the average length of the chain segment is minimal. In DMSO, due to high polarity and solvation, the rate of crosslink formation decreases, so the M_C_ increases. In the case of DEG (a viscous medium with the formation of intense hydrogen bonds), the diffusion of radicals and monomers is complicated, so a chain with a higher molecular weight is formed between the crosslinking nodes. In cyclohexanol, which is less polar and more viscous, the probability of functional groups contact is even lower, and the distance between crosslinking nodes is maximum.

The choice of solvent significantly affects the mobility of PVP and HEMA, and, consequently, the grafting efficiency and the crosslinking density. Polar solvents (H_2_O, DMSO) provide better radical mobility, but promote HEMA homopolymerization, while more viscous or less polar media (DEG, HOCy) limit the frequency of crosslink formation and increase the average length of the polymer segment between them.

**Figure 6:**
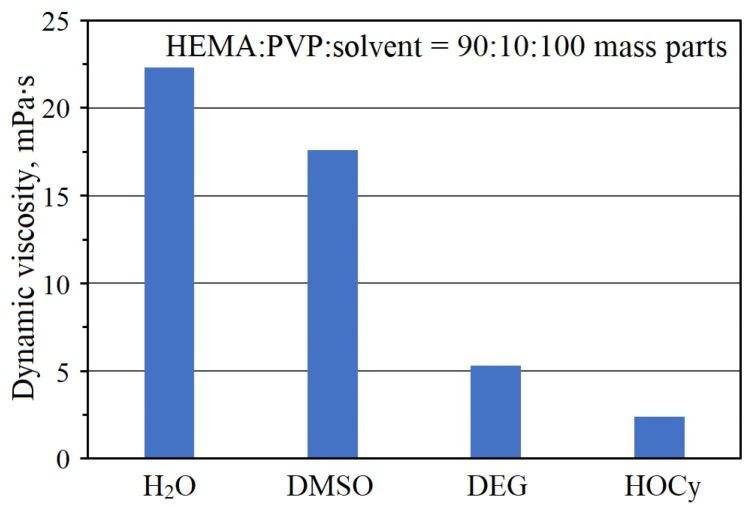
Influence of the solvent’s nature on the dynamic viscosity of HEMA/PVP compositions (without initiator).

The interaction of HEMA/PVP compositions with solvents is confirmed by the study of their dynamic viscosity. As shown in Figure 6, it was established that the dynamic viscosity of MPC does not correlate directly with the viscosity of a pure solvent in the presence of a solvent (Table 2). The viscosity of the composition decreases in the series DEG–HOCy–H_2_O–DMSO depending on the solvent’s nature. As can be seen, the viscosity of the pure solvent is not the main factor affecting the viscosity of the initial MPC solution. The viscosity of the MPC solution is also dependent on the solvating ability of the solvent. The minimum viscosity of the composition in DMSO is explained by its high efficiency as a solvent for PVP. DMSO is an excellent aprotic solvent for both HEMA and PVP. It effectively solvates PVP, leading to the formation of compact polymer tangles. The compactness of PVP tangles means they have a small hydrodynamic volume and, therefore, minimally affect the increase in solution viscosity compared to pure solvent. Consequently, the composition’s viscosity remains almost unchanged in comparison to the viscosity of pure DMSO.

The viscosity of MPC increases sharply in water and is higher than in DMSO. In water, PVP and HEMA are prone to hydrophobic associations and the formation of a strong hydrogen bond network.

At the same time, the high viscosity of the DEG composition is due to the combination of DEG’s own high viscosity as a solvent and its strong association with PVP. DEG contains two OH groups that can form strong hydrogen bonds with the C=O group of PVP. Unlike DMSO, DEG can promote the unwinding of PVP polymer coils and strengthen their interaction with HEMA, as well as solvating. Using HOCy as a solvent results in the most significant decrease in the viscosity of MPC. Despite its high viscosity (Table 1), HOCy demonstrates the lowest polarity and the lowest ability to form strong hydrogen bonds with the polar PVP and HEMA. Therefore, in the HOCy environment, PVP acquires a compact (folded) conformation, resulting in minimal effects on viscosity.

Based on the results presented, it can be concluded that the viscosity of the medium has a significant impact on the mobility of macromolecules and, consequently, on the formation of the polymer network structure of pHEMA-gr-PVP copolymers.

Thus, by using different contents and nature of solvents, it is possible to widely regulate the composition of the copolymer and the density of the polymer network. Subsequently, this influences the properties of the polymers.

### 2.2. The Properties of pHEMA-gr-PVP Copolymers

**Figure 7:**
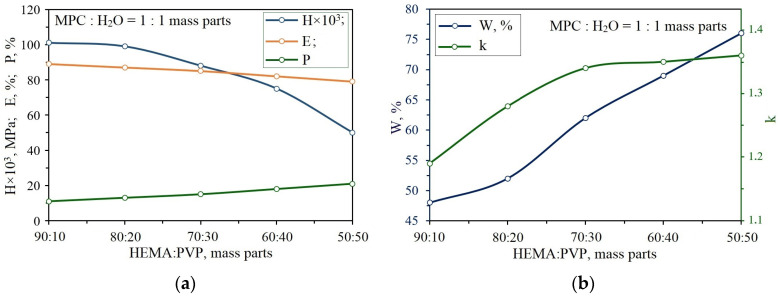
The influence of the composition of the MPC on the deformation–elastic (**a**) and sorption (**b**) characteristics of pHEMA-gr-PVP copolymers.

To characterize the properties of the synthesized hydrogels, their hardness, elasticity, plasticity, and ability to swell in solvents were investigated. The hardness, elasticity, and plasticity of hydrogels were characterized, respectively, by the hardness number (H, MPa), elasticity number (E, %), and plasticity number (P, %). The swelling ability was characterized by such parameters as boundary water absorption (W, %) and hydrogel swelling factor (k). Hardness, elasticity, and plasticity determine the mechanical and elastic characteristics of hydrogels during operation; the ability to swell determines such properties as elasticity, biotolerance, and permeability for low molecular weight substances. The study results of the influence of the composition formulation on the physico-mechanical properties of hydrogels are shown in Figure 7.

Increasing the PVP content in the starting MPC reduces the hardness and elasticity of the copolymers (Figure 7a). This is apparently due to the fact that during hydration, part of the PVP macromolecules is washed out of the copolymer and does not undergo the applied load. As a result, the lower the grafting efficiency of PVP macromolecules, the more porous the polymer and, as a result, the lower the hardness and elasticity. This is also the main reason, along with the hydrophilicization of the network, for the increased water absorption of the solvent by the copolymers (Figure 7b).

**Figure 8:**
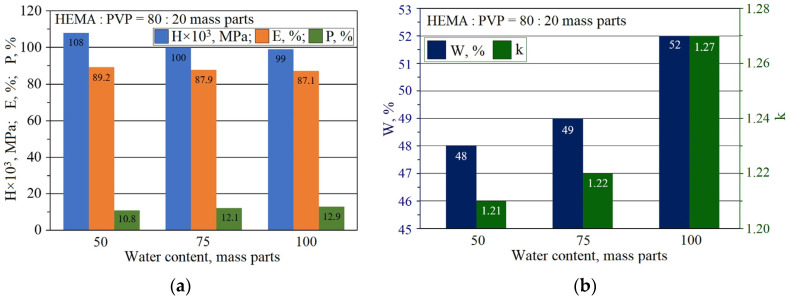
Effect of solvent content on the deformation–elastic (**a**) and sorption (**b**) characteristics of pHEMA-gr-PVP copolymers.

With increasing water content in the starting composition, hydrogels with lower crosslinking density are formed (Figure 4), which naturally causes a decrease in hardness and elasticity and a simultaneous increase in water content and swelling coefficient in the final hydrogel (Figure 8).

As the M_C_ increases, the polymer network becomes less dense. Such a structure loses rigidity, so the material is characterized by lower hardness and elasticity numbers. At the same time, the more loosely crosslinked polymer network possesses larger free volumes and cavities allowing water molecules to easily penetrate into it. This causes an increase in the equilibrium water content of the hydrogel. The obtained result corresponds to the classical ideas about the relationship between the structural parameters of the polymer network and the physico-mechanical characteristics of hydrogels.

**Figure 9:**
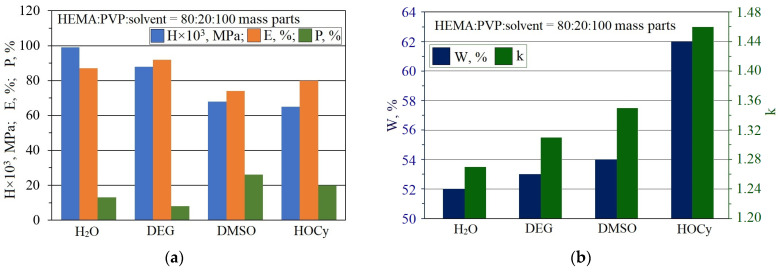
Effect of the nature of the solvent on deformation–elastic (**a**) and sorption (**b**) characteristics of pHEMA-gr-PVP copolymers.

Upon analyzing the influence of the solvent nature (Figure 9), it can be noted that the values of hardness and elasticity when using water and DEG are the highest. At the same time, in the case of DEG, there is also a slight increase in water absorption and swelling coefficient.

Analysis of the dependence of M_C_ on the nature of the solvent (Figure 5b) with experimental data on the physico-mechanical characteristics of hydrogels (Figure 9) indicates their relationship. In the series H_2_O, DEG, DMSO, HOCy, a gradual increase in M_C_ is observed. When water is used, the densest polymer network is formed with a high frequency of crosslinking nodes, which provides relatively high hardness and elasticity numbers and low water content and swelling coefficient values. Switching to less polar or more viscous solvents leads to a decrease in the rate of crosslinking node formation, as a result of which the polymer network becomes less dense and the average chain length between crosslinks increases. This is naturally reflected in a decrease in the strength and elastic characteristics but an increase in the water-retention capacity of hydrogels. The most pronounced effect is observed in the case of cyclohexanol, where due to its having the lowest polarity and high viscosity, conditions are created for the formation of the least crosslinked spatial network, which is reflected in the minimum hardness and elasticity numbers, but the maximum values of water content and swelling coefficient.

Thus, the established structure–function relationship confirms that, with an increase in the molecular weight of the polymer chain segment between crosslinking nodes, the hydrogels lose strength and elasticity, while their ability to hydrate and swell increases significantly. The use of a solvent during polymerization is a powerful tool for predictably controlling the mechanical properties of hydrogels. This allows to create materials with specified characteristics that mimic the properties of biological tissues or meet the specific requirements of medical devices.

## 3. Conclusions

The effect of the amount and nature of the solvent on the structure and mechanical properties of hydrogels based on copolymers of HEMA with PVP was studied. Dilution of the monomer–polymer composition with a solvent allowed to increase the maximum PVP content in the starting mixture to 50 mass parts, which is impossible during bulk polymerization due to high viscosity. An increase in the amount of solvent (water) to 50 mass parts increased the efficiency of PVP grafting and its content in the final copolymer, which is associated with the dilution of the MPC (reduction in viscosity) and an improvement in the mobility of macromolecules.

It was found that the grafting efficiency and the PVP content in the copolymer structure decrease in the series DEG, H_2_O, DMSO, which is explained by the different polarity and viscosity of the solvents, where DEG provides intensive hydrogen bonds with PVP, and DMSO promotes the homopolymerization of HEMA. Polar solvents (H_2_O, DMSO) provide better radical mobility, but promote homopolymerization of HEMA, while more viscous or less polar media (DEG, HOCy) limit the frequency of crosslinking node formation and increase the average length of the polymer segment between them. It was found that with an increase in the molecular weight between crosslinks in the series H_2_O, DEG, DMSO, HOCy, the polymer network becomes less dense, which is reflected in a decrease in the mechanical and elastic characteristics of hydrogels and a simultaneous increase in their water-retaining capacity and swelling coefficient. This indicates a direct structure–function relationship between the nature of the solvent, network parameters, and the performance properties of hydrogels.

It has been shown that the choice of the nature and concentration of the solvent is a powerful tool for the predictive control of the copolymer formulation and the density of the polymer network, which allows to create hydrogels with the necessary mechanical and sorption characteristics for biomedical applications.

## 4. Materials and Methods

### 4.1. Materials

The following reagents were used in the research:

– 2-hydroxyethylmethacrylate (Sigma Chemical Co., Saint Louis, MO, USA) after purification via vacuum distillation was conducted at a residual pressure of 130 N/m^2^ and a boiling temperature of 351 K;
– Polyvinylpyrrolidone (AppliChem GmbH, Darmstadt, Germany) with a molecular weight of 28,000 was dried under vacuum at 338 K for 2–3 h prior to its application;
– Iron (II) sulfate (FeSO_4_) was employed as pro analysis (p.a.) grade reagent.

### 4.2. pHEMA-gr-PVP Copolymer Synthesis

pHEMA-gr-PVP copolymers were obtained at ambient temperature (20–25 °C). The reaction, catalyzed by FeSO_4_, proceeded for a duration varying from 0.3 to 1.5 h, the exact time being dictated by the specific composition formulation.

**Figure 10:**
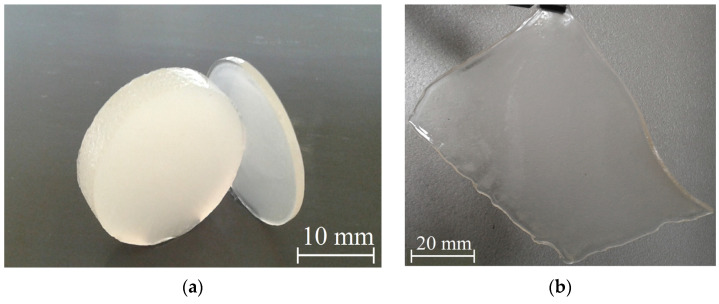
Experimental hydrogel samples obtained by the method of pouring into a mold (**a**) and rotational molding (**b**).

Following the recipe, the assessed amounts of PVP and FeSO_4_ were weighed, and HEMA and H_2_O were dosed by the volumetric method. The required amount of PVP was dissolved in HEMA, while FeSO_4_ (0.05 wt.%) was dissolved in water. Stirring was carried out at room temperature until PVP was completely dissolved in HEMA and FeSO_4_ in water. An aqueous solution of FeSO_4_ and a solution of PVP in HEMA were mixed to obtain a homogeneous composition, free of insoluble agglomerates and mechanical inclusions. After it was processed by pouring into a polymerization mold (Figure 10a) or by rotational molding [23] (Figure 10b). The resulting products were washed in distilled water until unreacted HEMA and PVP were completely removed.

### 4.3. Measurements and Characterization

#### 4.3.1. Standard Methods of Instrumental Research

Attenuated total reflectance Fourier transform infrared (ATR FTIR) spectra were obtained in accordance with the method presented in [37,38].

The morphology of the hydrogels in the swollen state was studied under low vacuum conditions in accordance with the method presented in [28].

#### 4.3.2. Measurement of the Dynamic Viscosity of Solutions

The measurement of dynamic viscosity of solutions was carried out according to the method described in ISO 3219: “Plastics—Polymers/resins in the liquid state or as emulsions or dispersions—Determination of viscosity using a rotational viscometer with defined shear rate” [49]. Dynamic viscosity was measured using a Rheostress RS75 rheometer (Haake, Thermo Electron GmbH, Karlsruhe, Germany) at a constant temperature of 20 ± 0.1 °C and a shear rate of 50 s^−1^. Measurements were taken using a coaxial cylinder measuring system (Couette geometry DG 25).

#### 4.3.3. Efficiency of PVP Grafting

The amount of unbound PVP in the polymer network was determined by photocolorimetry of the aqueous extract, using the property of PVP to form a colored complex with iodine [50].

The efficiency of grafting (f, %) was determined by calculating the ratio of the mass of grafted polyvinylpyrrolidone (m_1_, g) to the total mass of polyvinylpyrrolidone introduced into the initial reaction mixture (m, g) [38]:

(1) $$f=\frac{m_{1}}{m}\cdot 100$$

The grafting degree (p, %) was calculated as a ratio between grafted PVP (m_1_, g) and the total weight of the copolymer (M, g):

(2) $$p=\frac{m_{1}}{M}\cdot 100$$

The measurement results were taken as the arithmetic mean of the results of testing 5 samples of each composition. The statistical error of the experiment is no more than 4%.

#### 4.3.4. Structural Parameters of the Polymer Network

The structural parameters of the polymer network were evaluated. by molecular weight between crosslinks in the polymer network (M_C_, kg/mol) and crosslinking density (μ, mol/m^3^), that were determined by the equilibrium modulus of high elasticity using the Flory–Rehner method [51].

In particular, M_C_ was determined [38] via the formula:

(3) $$E=3\frac{\mathrm{\rho RT}}{M_{C}}$$

M_C_ represents the molecular weight between the cross-links in the polymer network (kg/mol); E is the equilibrium high elasticity modulus (N/m^2^); ρ is the density of the polymer (kg/m^3^); T is the temperature at which the tests were conducted (K); and R is the universal gas constant (J/mol∙K).

For the research, it samples with sizes of 8 mm in height and a 15 mm diameter were used. Before testing, these samples were hydrated to their equilibrium state, followed by extraction to ensure the complete removal of any non-grafted PVP.

Mechanical tests were executed on a Hepler consistometer (VEB Pruefgeraetewerk, Medingen, Dresden, Germany). The samples were subjected to constant deformation, never exceeding 30%, under varying compressive loads.

Applying the data obtained, it is possible to construct a plot showing the dependence of equilibrium high elasticity deformation ε (%) versus the applied compressive stress (kgf/cm^2^). The equilibrium modulus of high elasticity (E) was subsequently derived from the slope angle (α) of the resulting linear relationship σ_i_ = f (ε_i_):

(4) E=tgα

The obtained value of E was then used to calculate the molecular weight between cross-links (M_C_) utilizing Formula (3). Finally, M_C_ served as the basis for calculating the crosslinking degree (5) of the polymer network.

(5) $$\mu =\frac{\rho }{2M_{C}}$$

The measurement results were taken as the arithmetic mean of the results of testing 5 samples of each composition. The statistical error of the experiment is no more than 4%.

#### 4.3.5. Physico-Mechanical Characteristics of pHEMA-gr-PVP Copolymers

To determine boundary water absorption (W, wt.%) and hydrogel swelling factor (k), the disc-shaped samples ∅15 mm (or films with dimensions 15 × 15 mm) were used with thickness 0.5 mm. The samples were hydrated in distilled water to equilibrium for 24 h.

Boundary water absorption (W, wt.%) was obtained as the difference between dry (m_0_, g) and swollen (m_1_, g) samples [38]:

(6) $$W=\frac{m_{1}-m_{0}}{m_{1}}\cdot 100$$

Hydrogel swelling factor was determined as the ratio of the dimensions of dry (d_d_, mm) and swollen (d_s_, mm) samples:

(7) $$k=\frac{d_{s}}{d_{d}},$$

The dimensions of the sample in dry and hydrated state were measured using an E5163.00 Cathetometer (Eberbach, Van Buren Charter Township, MI, USA). Accuracy of measurements is ±0.01 mm.

The result of the measurements was taken as the arithmetic mean of 5 samples. The statistical error of the experiment is no more than 3%.

Deformation and elastic characteristics of samples in swollen state, were determined according to the procedures described in and ASTM D2240-15 “Standard Test Method for Rubber Property–Durometer Hardness” [52], using a TShR-320 hardness meter (JSC «Polymermash», Saint Petersburg, Russia), by measuring a difference between the depths of indenter penetration under the action of previous and residual loads [36].

Cylindrical samples with a diameter of 20 mm and a thickness of 7 mm were used for the research. The applied force was 0.6 N. The load was applied smoothly, without jerks. The depth of indentation (h) was measured without removing the load, 60 ± 5 s after applying the full load. The depth of residual deformation (h_1_) (to determine the plasticity number) was measured 60 ± 5 s after the load was completely removed. The samples were loaded and unloaded within 30 ± 2 s.

Hardness number (H, MPa), elasticity index (E, %), and plasticity index (P, %) were determined by the following formulas:

(8) $$H=\frac{0.1F}{\pi \cdot d\cdot h}$$

(9) $$E=\frac{h-h_{1}}{h}\cdot 100$$

(10) $$P=\frac{h}{h_{1}}\cdot 100$$

where: F is applied load, N (0.6 N); d is diameter of indenter ball, mm (d = 5 mm); h is depth of ball penetration into the sample (after 60 ± 5 s) under the load F, mm; h_1_ is residual deformation after removing the load (after 60 ± 5 s), mm.

The results of the measurements were taken as the arithmetic mean of the hardness, elasticity, and plasticity of 5 samples. The statistical error of the experiment is no more than 4%.

## Figures and Tables

**Table 1:** Results of studying the effect of the HEMA:PVP ratio in MPC on the structural parameters of the polymer network (MPC:H_2_O = 1:1 mass parts).

№	HEMA	PVP	E, kgf/cm^2^	M_C_, kg/mol	ρ, kg/m^3^	μ, mol/m^3^
1	100	0	5.41	12	1308	54.08
2	90	10	4.45	21	1295	30.86
3	80	20	3.65	26	1288	24.77
4	70	30	3.35	28	1260	22.50
5	60	40	2.80	34	1210	17.79
6	50	50	1.50	63	1135	9.00

**E** is equilibrium modulus of high elasticity; **M_C_** is molecular weight between cross-links in the polymer network; **ρ** is polymer density; **μ** is crosslinking density.

**Table 2:** Physical and chemical characteristics of solvents [47,48].

Solvent	Dynamic Viscosity, mPa⋅s	Polar Index	Solvent Type	Solvation Capacity
H_2_O	0.89	10.2	Protic	Strong donor and acceptor of H-bonds.
DMSO	1.99	7.2	Aprotic	Strong H-bond acceptor. Solvates cations and polar molecules.
DEG	35.7	7.5	Protic	Strong donor and acceptor of H-bonds.
HOCy	41.1	4.0–5.0	Protic	Less polar than H_2_O or DEG. Has a significant nonpolar fragment.

## Data Availability

The original contributions presented in the study are included in the article, further inquiries can be directed to the corresponding author.

## Abbreviations

The following abbreviations are used in this manuscript:

HEMA	2-hydroxyethylmethacrylate
PVP	Polyvinylpyrrolidone
DMSO	Dimethyl sulfoxide
DEG	Diethylene glycol
HOCy	Cyclohexanol
MPC	Monomer–polymer composition
